# SRT2183 impairs ovarian cancer by facilitating autophagy

**DOI:** 10.18632/aging.104126

**Published:** 2020-11-20

**Authors:** Tingting Sun, Yanfen Hu, Weipeng He, Yuru Shang, Xiaohong Yang, Liyun Gong, Xianbin Zhang, Peng Gong, Guofen Yang

**Affiliations:** 1 Department of Gynecology, First Affiliated Hospital of Sun Yat-sen University, Guangzhou 510080, China; 2 Discovery Department, Elpiscience Biopharma Ltd., Shanghai 201203, China; 3 Department of Plastic Surgery, Shenzhen University General Hospital and Shenzhen University Clinical Medical Academy, Shenzhen 518055, China; 4 Department of Biochemistry and Molecular Biology, Shenzhen University Health Science Center, Shenzhen 518060, China; 5 Department of General Surgery and Carson International Cancer Research Center, Shenzhen University General Hospital and Shenzhen University Clinical Medical Academy, Shenzhen 518055, China; 6 Guangdong Key Laboratory for Biomedical Measurements and Ultrasound Imaging, School of Biomedical Engineering, Shenzhen University Health Science Center, Shenzhen 518060, China; 7 Guangdong Key Laboratory of Regional Immunity and Diseases, Shenzhen University Health Science Center, Shenzhen 518060, China

**Keywords:** STR2183, autophagy, apoptosis, AKT/mTOR pathway, p38 MAPK pathway

## Abstract

The 5-year survival rate of ovarian cancer patients is only 47%, and developing novel drugs for ovarian cancer is needed. Herein, we evaluated if and how SRT2183, a sirtuin-1 activator, impairs the ovarian cancer cells. OVCAR-3 and A2780 cells were treated with SRT2183. Cell viability was measured by cell counting kit-8 assay and clonogenic assay. Apoptosis was determined by flow cytometry with Annexin V and propidium iodide. The level of autophagy was evaluated by western blot and immunofluorescence. The activities of AKT/mTOR/70s6k and MAPK signaling pathway were measured by immunoblot. SRT2183 inhibited the growth of ovarian cancer cells, increased the accumulation of BAX, cleaved-caspase 3 and cleaved-PARP, and decreased the level of anti-apoptotic Bcl-2 and Mcl-1. SRT2183 increased the LC3II level, and enhanced the degradation of p62/SQSTM1. SRT2183 increased the formation of GFP-LC3 puncta and induced the maturation of autophagosome. Interestingly, knockdown of autophagy related 5 and 7 significantly impaired the anti-carcinoma activity of SRT2183, implying that SRT2183 impaired the ovarian cancer cells by inducing autophagy. SRT2183 decreased the accumulation of p-Akt, p-mTOR and p-70s6k, and activated the p38 MAPK signaling pathway. This indicated that Akt/mTOR/70s6k and p38 MAPK signaling pathway might be involved in the SRT2183-mediated autophagy and apoptosis.

## INTRODUCTION

Ovarian cancer is the second most common cancer of the female reproductive system and has the highest mortality rate in female reproductive disease [1]. Although improvements have been achieved in surgery [2], chemotherapy [3, 4], and immunotherapy [5, 6], the prognosis of ovarian cancer still remains poor [7]. Therefore, it is urgent to develop novel therapies for ovarian cancer patients.

SRT2183 has been demonstrated to inhibit cell growth and induce cellular demise of malignant lymphoid, glioblastoma and malignant bone tumor [8–10]. However, it remains unclear whether SRT2183 impairs the ovarian cancer cells.

Autophagy can degrade dysfunctional organelles and damaged proteins, and plays a critical role in maintaining the survival of cells [11, 12]. Previous studies proved that autophagy was involved in cell apoptosis [13–15]. For example, Wang et al. demonstrated that inhibition of autophagy could promote apoptosis [14]. However, Yu et al. suggested that inhibition of autophagy could reduce the level of apoptosis [16]. This suggests an ambivalent function of autophagy in apoptosis. In addition, previous studies reported that the autophagy could be regulated by Akt/mTOR/70s6k signaling or MAPK signaling pathway [17–20]. Thus, in this study, we evaluated whether SRT2183 was a promising chemotherapy for ovarian cancer cells. In addition, we investigated if and how the toxicity effect of SRT2183 was regulated by autophagy. Moreover, we determined if the traditional autophagic signaling pathway (Akt/mTOR/70s6k signaling pathway and the MAPK pathway) were involved in the SRT2183 mediated autophagy.

## RESULTS

### SRT2183 inhibits proliferation of ovarian cancer cells and induces apoptosis

**Figure 1:**
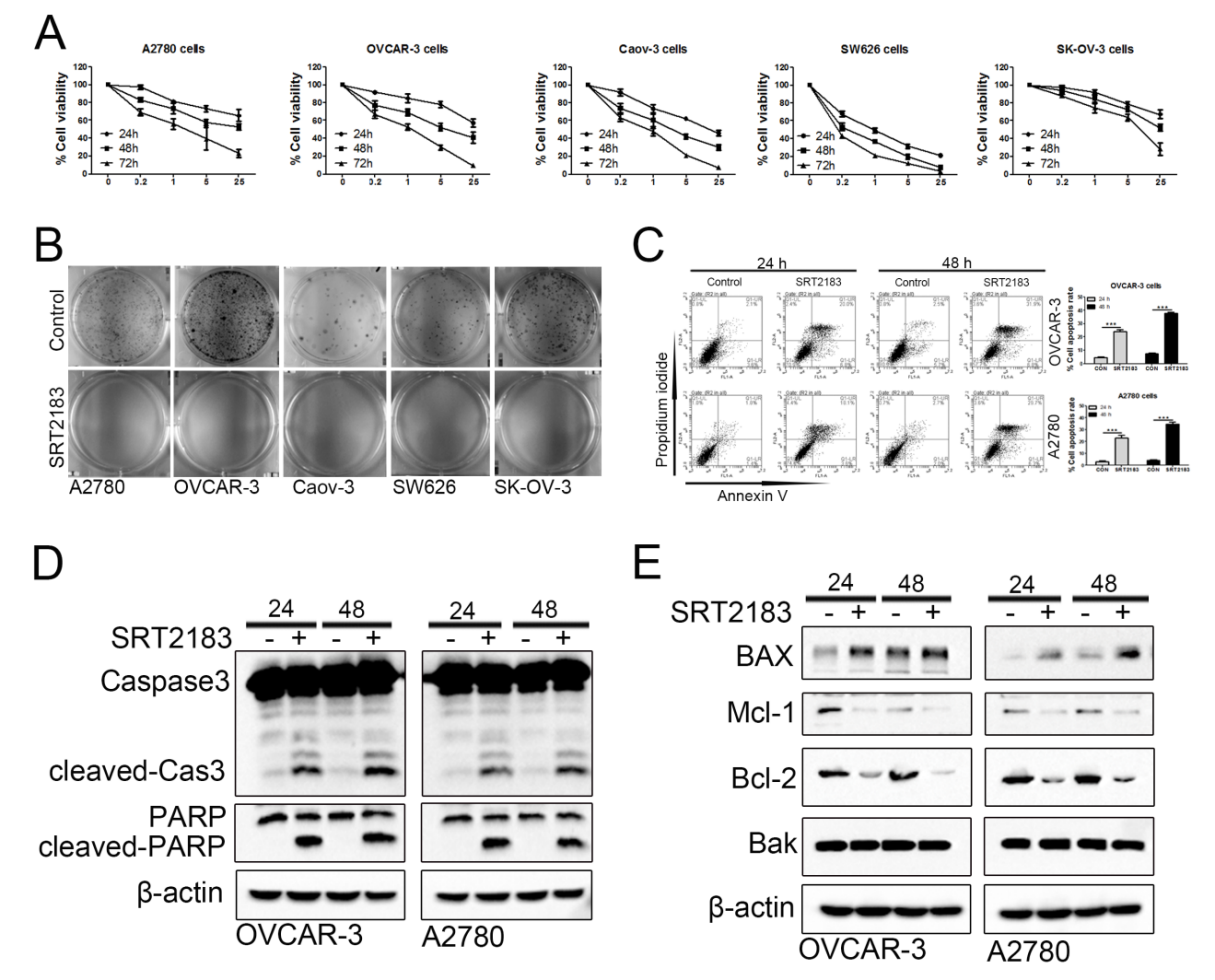
**SRT2183 suppresses cell proliferation and triggers apoptosis in ovarian cancer cells.** (**A**) A2780, OVCAR-3, Caov-3, SW626, and SK-OV-3 cells were treated with vehicle or SRT2183 (0.2, 1, 5, and 25 μM) for 24, 48, and 72 h, the cell proliferation was detected by CCK8 assay. (**B**) A2780, OVCAR-3, Caov-3, SW626, and SK-OV-3 cells were treated with vehicle or 1 μM SRT2183 for 14 days, the cell growth was determined by colony formation analysis. (**C**) OVCAR-3 and A2780 cells were treated with vehicle or 1 μM SRT2183 for 24 and 48 h, cell apoptosis was detected with the help of Annexin V, propidium iodide staining and flow cytometry. (**D**) OVCAR-3 and A2780 cells were treated by the same procedures described in (**C**), the expression of cleaved-Caspase-3 (cleaved-Cas3), and cleaved-PARP was detected by immunoblot analysis. (**E**) OVCAR-3 and A2780 cells were treated by the same procedures described in (**C**), the expression of BAX, Mcl-1, Bcl-2 and Bak was detected by western blot. N=3 for (**A**), (**B**), (**D**) and (**E**); N=5 for (**C**); *** indicates *P* < 0.001.

In order to evaluate if SRT2183 impairs ovarian cancer cells, CCK8 assay was performed. We observed that SRT2183 decreased the cell viability when compared to vehicle-treated cells in a dose- and time- dependent manner (Figure 1A). In addition, the colon assay demonstrated that 1 μM SRT2183 inhibited the growth of A2780, OVCAR-3, Caov-3, SW626, and SK-OV-3 cells (Figure 1B). To evaluate if and how SRT2183 regulates apoptosis, the OVCAR-3 and A2780 cells were incubated with 1 μM SRT2183 for 24 and 48 h, and the apoptosis was determined by FACS with the help of Annexin V and propidium iodide staining. We observed that compared to vehicle-treated cells, SRT2183 significantly increased the apoptosis of OVCAR-3 and A2780 cells after these cells were incubated with SRT2183 for 24 h or 48 h (Figure 1C). In addition, the immunology analysis demonstrated that 1 μM SRT2183 increased the level of two transactional apoptotic proteins: cleaved-Caspase3 (cleaved-Cas3) and cleaved-PARP (Figure 1D). In order to evaluate how SRT2183 induces apoptosis, the level of pro-apoptotic proteins such as BAX and Bak, and anti-apoptotic proteins such as Mcl-1 and Bcl-2, was determined. The immunology assay proved that SRT2183 increased the level of BAX, whereas it failed to regulate the level of Bak (Figure 1E). Meanwhile, SRT2183 decreased the level of MCl-1 and Bcl-2 (Figure 1E). Taken together, these results suggested that SRT2183 inhibited the proliferation and induced the apoptosis of ovarian cancer cells.

### SRT2183 induces autophagy

**Figure 2:**
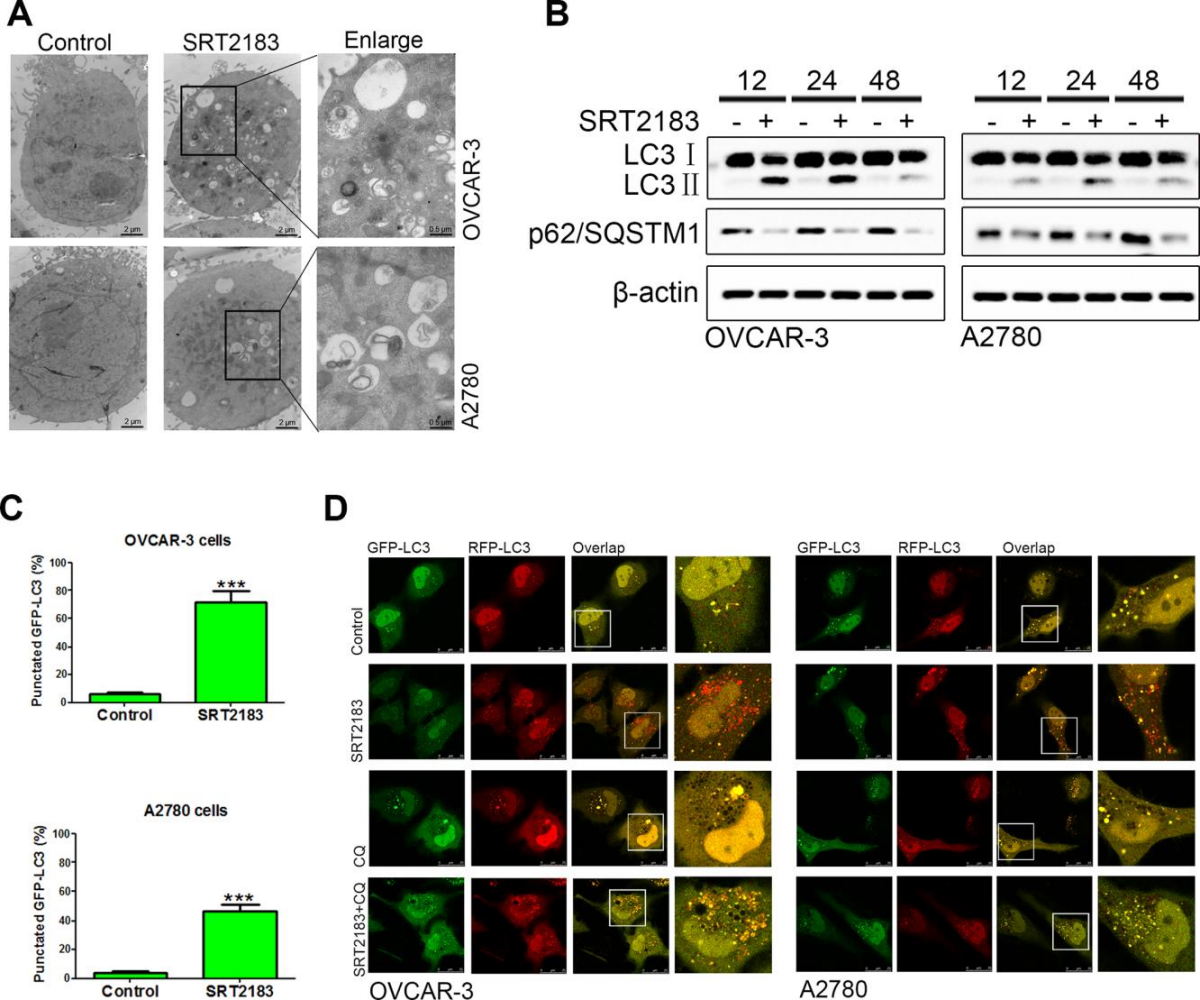
**SRT2183 induces autophagy in ovarian cancer cells.** (**A**) OVCAR-3 and A2780 cells were treated with vehicle or 1 μM SRT2183, then observed by transmission electron micrograph (TEM). (**B**) After incubation with 1 μM SRT2183, the expression of LC3 and p62/ SQSTM1 was detected by western blot. (**C**) OVCAR-3 and A2780 cells were pre-transfected with GFP-LC3, after 24 h the cells were treated with vehicle or 1 μM SRT2183 for 24 h. The LC3 was observed by confocal microscope. (**D**) OVCAR-3 and A2780 cells were transfected with GFP- RFP-LC3 for 24 h and pre-treated with chloroquine (CQ) for 6 h, then the cells were treated with vehicle or 1 μM SRT2183 for 24 h. Images were obtained by a confocal microscope. Red dot (GFP−, RFP+): autolysosomes; yellow dot (GFP+, RFP+): autophagosome. N=3 for (**A**), (**B**) and (**D**). N=6 for (**C**), *** indicates *P* < 0.001.

In order to evaluate if and how SRT2183 regulates the level of autophagy, the OVCAR-3 and A2780 cells were incubated by 1 μM SRT2183 and the autophagosome was evaluated by transmission electron microscopy. We observed that SRT2183 decreased the level of autophagosomes, when compared to the cells treated by vehicle (Figure 2A). In addition, the level of autophagy was evaluated by LC3 and p62/SQSTM1 western blot. We found that SRT2183 increased the level of LC3II, however, it decreased the accumulation of p62/SQSTM1 (Figure 2B). In addition, the SRT2183-treated cells induced a significant increase in microscopy-based GFP-LC3 puncta, when compared to vehicle cells (Figure 2C). Moreover, compared to vehicle-treated cells, 1 μM SRT2183-treated cells had more red dots (autolysosomes, Figure 2D). As expected, adding 5 μM chloroquine (CQ), a traditional inhibitor of autophagy, increased the number of yellow dots (autophagosomes, Figure 2D) in OVCAR-3 and A2780 cells. Taken together, these results indicated that SRT2183 induced autophagy in ovarian cancer cells.

### Autophagy is involved in the SRT2183-mediated anti-carcinoma effect

**Figure 3:**
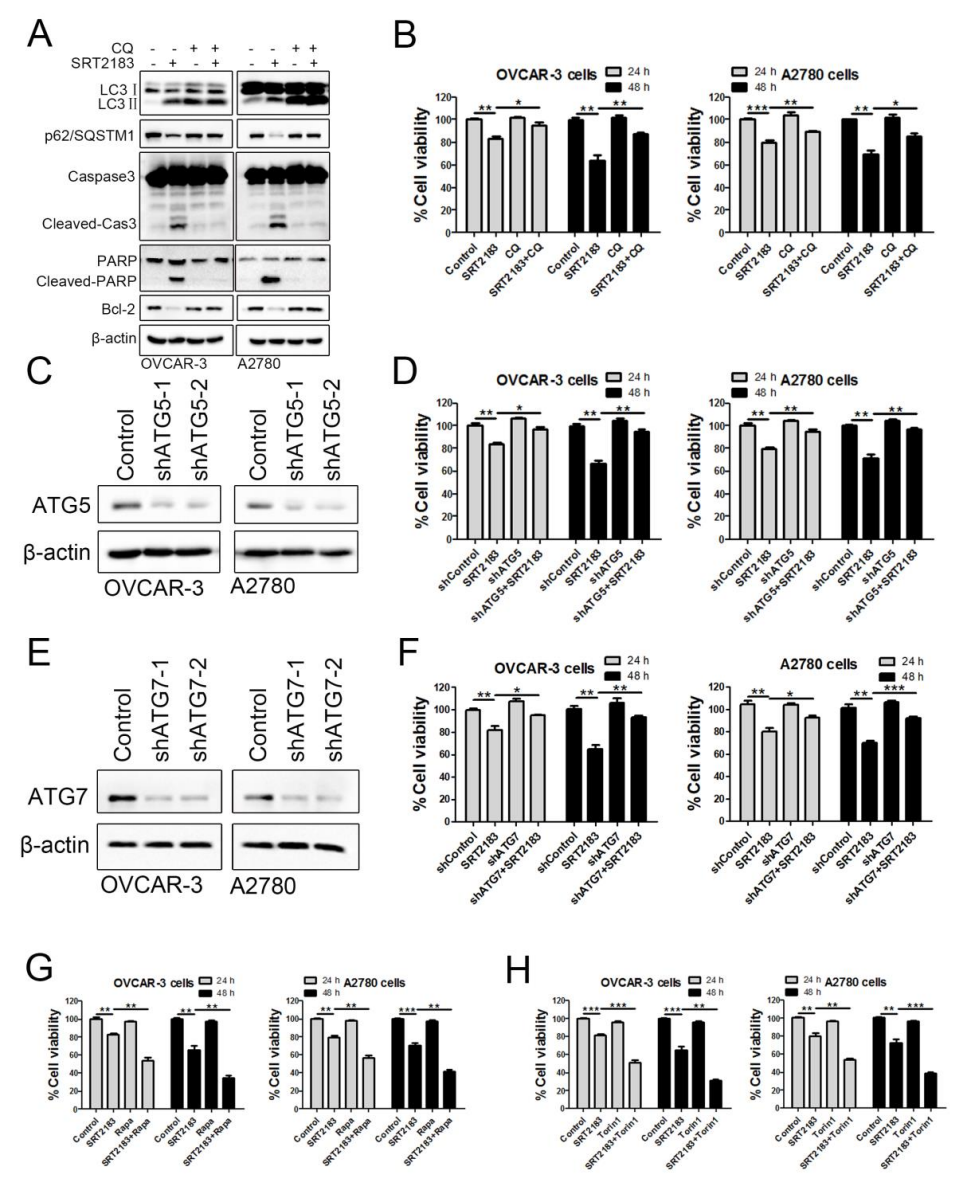
**The anti-carcinoma activity of SRT2183 is dependent on autophagy.** (**A**) OVCAR-3 and A2780 cells were pre-treated with chloroquine (CQ) for 6 h, then treated with vehicle or 1 μM SRT2183. The expression of LC3, p62/SQSTM1, Caspase3, PARP and Bcl-2 was detected by western blot. (**B**) OVCAR-3 and A2780 cells were treated by the same procedure described in (A) for 24 and 48 h, the viability of OVCAR-3 and A2780 cells were detected by CCK8 assay. (**C**) The expression of autophagy related 5 (ATG5) was knocked down by shRNA-lentivirus (shATG5-1 and shATG5-2) and the protein was determined by western blot. (**D**) OVCAR-3 and A2780 cells were treated by vehicle, shATG5-1, 1 μM SRT218 or the combination of shATG5-1 and SRT218 for 24 and 48 h. The viability of the cells was evaluated by CCK8 assay. (**E**) The expression of autophagy related 5 (ATG5) was detected by western blot and (**F**) The cell viability was determined after the cells were treated by 1 μM SRT2183 for 24 and 48 h. (**G**, **H**) OVCAR-3 and A2780 cells were pre-treated with 0.1 μM Rapamycin (Rapa) or 0.05 μM Trion 1 for 6 h, then the cells were treated with vehicle or 1 μM SRT2183 for 24 and 48 h. The cell viability was detected by CCK8 assay. N=3 for (**A**) and (**C**); N=5 for (B), N=6 for (**D**), (**E**) and (**F**). * indicates *P* < 0.05; ** indicates *P* <0.01; *** indicates *P* < 0.001.

In order to evaluate if autophagy contributed to the SRT2183-mediated anti-carcinoma effect, the autophagy was blocked by 5μM CQ. We observed that compared to SRT2183, the combinational therapy of CQ plus SRT2183 increased the level of LC3II (Figure 3A), p62/SQSTM1 (Figure 3A) and autophagosomes (Figure 2D), and decreased the accumulation of autolysosomes (Figure 2D). This suggested that CQ successfully inhibited the SRT2183-mediated autophagy flux. In addition, we found that compared to the cells treated by SRT2183, the combinational therapy of CQ plus SRT2183 significantly decreased the level of pro-apoptotic protein, such as cleaved-Cas3 and cleaved-PARP. However, it increased the accumulation of anti- apoptotic protein, Bcl-2 (Figure 3A). This suggested that blocking autophagy reduced the SRT2183-mediated apoptosis. The CCK8 assay demonstrated that SRT2183 significantly decreased the viability of OVCAR-3 cells and A2780 cells when compared to the vehicle-treated cells (Figure 3B), whereas CQ did not significantly influence the cell viability (Figure 3B). Interestingly, we observed that CQ in combination with SRT2183 significantly enhanced the cell viability, when compared to SRT2183 treated cells (Figure 3B). These results implied that autophagy might be involved in the SRT2183-mediated apoptosis of OVCAR-3 cells and A2780 cells.

In order to verify if the anti-cancer effect of SRT2183 is dependent on the autophagy, we knocked down autophagy related 5 (ATG5) and autophagy related 7 (ATG7), two master regulators of autophagy, by shRNA-lentivirus. Indeed, the shRNA-lentivirus decreased the level of ATG5 in OVCAR-3 cells and A2780 cells (Figure 3C). Knocking down ATG5 in combination with SRT2183 significantly increased the cell viability when compared to SRT2183 treated cells (Figure 3D). Similar results were observed when the ATG7 was knocked down (Figure 3E, 3F). This suggested that SRT2183 inhibited cell growth by inducing autophagy. In order to justify if the induce of autophagy enhances the anti-proliferation effect of SRT2183, we treated OVCAR-3 and A2780 cells with 1 μM SRT2183, or the autophagy inducers (0.1 μM rapamycin or 0.05 μM torin 1), or the combinational therapy as indicated in Figure 3E, 3F. We observed that 0.1 μM rapamycin or 0.05 μM torin 1 did not significantly reduce the cell viability (Figure 3G, 3H).

However, rapamycin or torin 1 in combination with SRT2183 significantly promoted the anti-proliferation effect of SRT2183, when compared to SRT2183 treated cells (Figure 3G, 3H). Taken together, these results suggested that the anti-carcinoma effect of SRT2183 was dependent on the autophagy.

### Akt/mTOR/70s6k pathway and p38 MAPK signaling pathway might be involved in SRT2183-induced autophagy

**Figure 4:**
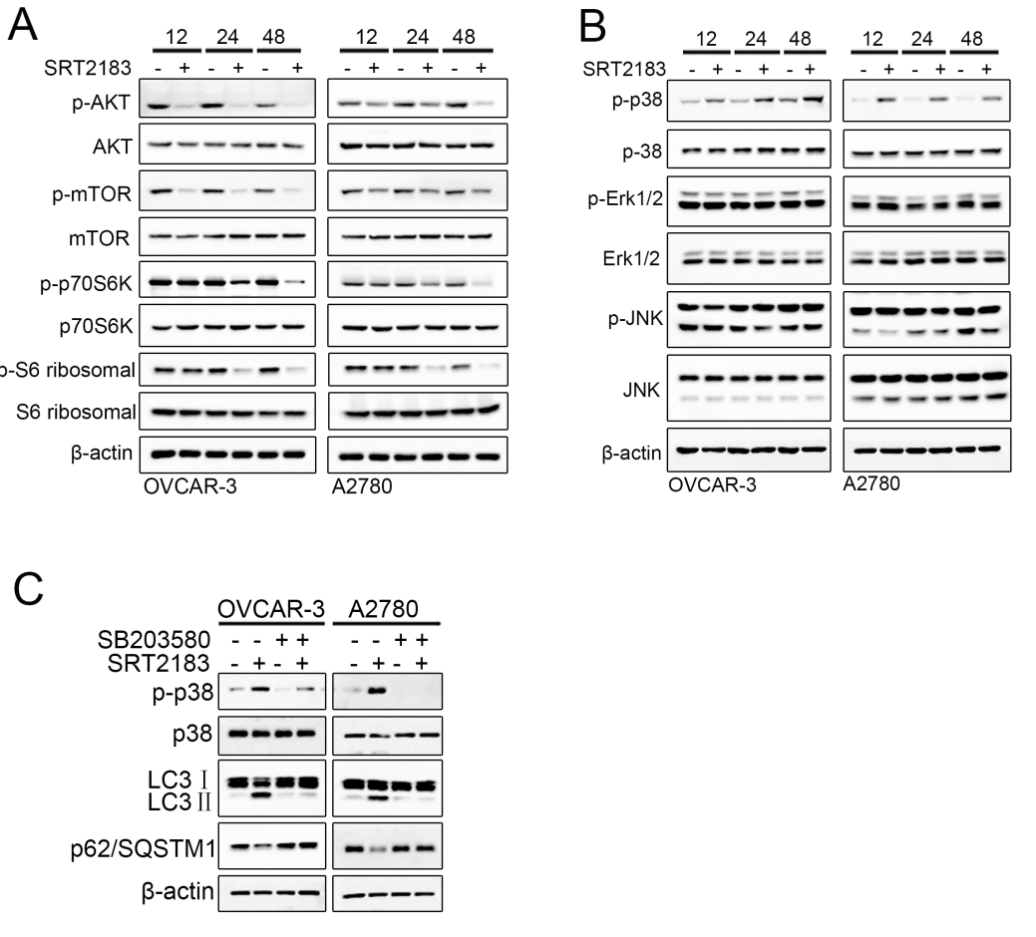
**SRT2183 inhibits AKT/mTOR/p70S6K pathway and actives p38 MAPK signaling pathway.** OVCAR-3 and A2780 cells were treated by 1 μM SRT218 for 12 h, 24 h, or 48 h. (**A**) The activities of AKT/mTOR/p70S6K pathway and (**B**) MAPK pathway were evaluated by western blot. (**C**) In order to evaluate if SRT2183 induce the autophagy by p38 signaling pathway, the cells were treated by SB203580, a traditional inhibitor of p38 MAPK signaling pathway. All experiments were repeated for three times.

In order to reveal the mechanism of how SRT2183 induces autophagy, we evaluated the Akt/mTOR/70s6k pathway and p38 MAPK signaling pathway, two traditional pathways of autophagy. A significant decrease in the phosphorylation of Akt, mTOR and p70S6K in both OVCAR-3 and A2780 cells was observed after these cells were treated by SRT2183 for 12 h, 24 h, or 48 h (Figure 4A). However, no significant decrease in Akt, mTOR, and p70S6K (Figure 4A) was observed. These results suggested that Akt/mTOR/70s6k pathway, a traditional signaling pathway of autophagy, was activated by SRT2183. To investigate if the MAPK signaling pathway is involved in the SRT2183-mediated autophagy, the expression level of p-p38, p-ERK and p-JNK was evaluated. SRT2183 increased the level of p-p38, whereas it did not influence the level of p-ERK and p-JNK (Figure 4B). In addition, the OVCAR-3 cells and A2780 cells were treated by 5 μM SB203580, a traditional inhibitor of p38 MAPK signaling pathway. We observed that compared to SRT2183, SB203580 in combination with SRT2183 inhibited the activity of p38 and theSRT2183-mediated autophagy (Figure 4C). This suggested that p38 MAPK signaling pathway might also be involved in the SRT2183- mediated autophagy.

## DISCUSSION

In this study, we observed that SRT2183 induced the apoptosis of ovarian cancer cells. This was supported by the observation that SRT2183 decreased the accumulation of anti-apoptotic protein Mcl-1 and Bcl-2 [21], and increased the level of BAX, a pro-apoptotic protein [22]. Previous study demonstrated that Bak was a master promoter of cell apoptosis [23]. The clustering of Bak on the mitochondrial outer membrane could lead to the transfer of pro-apoptotic proteins from mitochondria into cytosol and thus the induction of cell apoptosis [24]. However, no evidence showed that SRT2183 could regulate the level of Bak (Figure 1E). This suggested that the SRT2183-induced autophagy was independent of Bak.

In addition, we proved that SRT2183 enhanced apoptosis by inducing autophagy. This suggested that inducing autophagy might be a promising strategy for ovarian cancer treatment. However, several previous studies demonstrated that the inhibition of autophagy could also successfully inhibit the tumor growth [25, 26]. These contradictory results implied that autophagy might play dual role in different contexts [27]. Thus, the benefit of the treatments by inducing or inhibiting autophagy for ovarian cancer needs to be further investigated before clinical application. One promising strategy for evaluating the benefit of treatments that targeting autophagy is to use patient-derived xenograft (PDX) models [28]. Increasing evidence has shown that with the help of PDX, clinicians could accurately predict the benefit of the treatments [29].

In order to verify if SRT2183 regulates autophagy, we performed LC3/p62/SQSTM1 western blot, microscopy-based GFP-LC3 puncta, and the tandem fluorescent-tagged LC3 (mRFP-EGFP-LC3) assay, according to the guidelines for the use and interpretation of assays for monitoring autophagy [30]. Although all these experimental results suggested that SRT2183 induced autophagy, there were still some technical limitations [30]. For example, LC3 is not a specific protein, which is also involved in the non-autophagy structures [31]. Despite the minor limitations, all these analyses showed that SRT2183 increased the level of autophagy in OVCAR-3 and A2780 cells. In addition, we followed the suggestion of the guideline [24] and applied CQ, rapamycin, torin 1 to modify the SRT2183-induced autophagy. We observed that the inhibition of autophagy by CQ could significantly impair the SRT2183-mediated apoptosis. However, the increase of the autophagy by rapamycin and torin 1 could significantly increase the SRT2183-mediated apoptosis. This indicated that the pro-apoptotic effect of SRT2183 was dependent on autophagy. This hypothesis was supported by the experiments shown in Figure 3C, 3D, in which the ATG5 was knocked down.

To investigate how SRT2183 regulates autophagy, we performed western blot and evaluated the activity of two traditional signaling pathways of autophagy: Akt/mTOR/70s6k [17, 18] and MAPK signaling pathway [19]. We observed that SRT2183 inhibited the level of p-mTOR. Previous studies suggested that inhibiting of the mTOR activity could significantly decrease the level of autophagy [32, 33]. Thus, we speculated that SRT2183 induced the autophagy by inhibiting the activity of p-mTOR in ovarian cancer cells. This hypothesis has been evaluated in osteoclasts [9]. For example, Gurt et al. observed that SRT2183 could activate MAPK and down-regulate the activity of mTOR, and thus promote autophagy in osteoclasts [9]. In addition, this suggested that p38 MAPK signaling pathway might also be involved in the SRT2183-mediated autophagy. This was supported by the observation that SRT2183 increased the level of p-p38 (Figure 4B). However, the mechanism of how Akt/mTOR/70s6k and MAPK signaling pathway regulate the SRT2183-mediated autophagy needs to be further verified and evaluated.

In conclusion, in this study we found that SRT2183 significantly inhibited the proliferation of ovarian cancer cells and induced apoptosis. SRT2183 was observed to induce autophagy and blocking of autophagy could significantly inhibit the anti-carcinoma effect of SRT2183. This suggested that the anti-cancerous effect of SRT2183 was dependent on autophagy.

## MATERIALS AND METHODS

### Cell culture and reagents antibodies

Ovarian cancer cell lines OVCAR-3, Caov-3, SW626, and SK-OV-3 cell lines were purchased from American Type Culture Collection (Manassas, VA, USA). A2780 cell line was purchased from ECACC (UK). OVCAR-3 cells were cultured in RPMI-1640 Medium (ATCC, #30-2001) with 0.01 mg/ml bovine insulin and 20% fetal bovine serum (FBS, HyClone; GE Healthcare Life Science, Logan, UT, USA). Caov-3 cells were fostered in Dulbecco’s Modified Eagle’s Medium (DMEM, ATCC, #30-2001) with 10% FBS. SW626 cells were incubated in Leibovitz’s L-15 Medium (ATCC, #30-2008) with 10% FBS. SK-OV-3 cells were trained in McCoy’s 5a Medium (ATCC, #30-2007) with 10% fetal bovine serum. A2780 cells were planted in RPMI-1640 Medium (ATCC, #30-2001) containing 10% FBS. 100 U/mL penicillin and 100 μg/mL streptomycin were added to all mediums. A2780, OVCAR-3, Caov-3, and SK-OV-3 cells were maintained in an incubator at 37°C with 5% CO_2_. SW626 cells were kept in an incubator at 37°C. SRT2183 (#HY-19759), SB203580 (#HY-10256), and Torin 1 (#HY-13003) were obtained from Med Chem Express (USA), chloroquine (#C6628), and rapamycin (#V900930) were purchased from Sigma (USA).

### Cell counting kit-8, clonogenic assay, and Annexin V/propidium iodide staining

In order to evaluate the proliferation of cells, we performed cell counting kit-8 (CCK-8) assay. 5×10^3^ per well A2780, OVCAR-3, Caov-3, SW626, or SK-OV-3 cells were cultured in 96-well plates, and these cells were allowed to grow for 24 h. After that, these cells were incubated with 0.2 μM, 1 μM, 5 μM, or 25 μM SRT2183 for 24 h, 48 h, or 72 h. Then, the cells were incubated with 10 μL CCK-8 solution (code HY-K0301, Med Chem Express, USA) for 4 h. The absorbance was determined by a microplate spectrophotometer (Molecular Devices LLC, Sunnyvale, CA, USA) at 450 nm. In order to investigate the growth of cells, the clonogenic assay was performed as previously described [34]. Briefly, A2780, OVCAR-3, Caov-3, SW626, or SK-OV-3 cells were plated in 6 well chambers. After 24h, these cells were incubated with 1 μM SRT2183 for 2 weeks. Subsequently, the replicate clones (≥ 50 cells) were randomly counted. In order to evaluate the apoptosis of ovarian cancer cells, 6×10^5^ OVCAR-3 and A2780 cells were seeded in 6 cm dishes. After 24 h, these cells were treated by vehicle or SRT2183 for 24 h and 48 h. The percentage of apoptosis cells was determined by flow cytometric analysis with the help of Annexin V and propidium iodide (PI). The Annexin V and PI staining kit were obtained from KeyGEN BioTECH of China (KGA107).

### Transmission electron microscopy

In order to observe the autophagosome, 5×10^6^ OVCAR-3 or A2780 cells were seeded in 6 cm dishes for 24 h. Subsequently, these cells were treated by 1 μM SRT2183 for 24 h. The cells were then fixed in 2.5% glutaraldehyde and 1% osmium acid solution, and dehydrated in an alcohol gradient. Eventually, the cells were prepared for flat embedding in Epon 812 and observed under JEM-2000EX electron microscope.

### Lentivirus transfected cell lines

In order to knock down the ATG5 or ATG7 gene, the 293T cells were transfected with packaging plasmid (PSPAX and PMD2G) and ATG5-knockdown plasmid or ATG7-knockdown (GE Healthcare Life Science, Logan, UT, USA) or noncoding shRNA plasmid (GE Healthcare Life Science, Logan, UT, USA) for 48 h. Subsequently, the supernatants were collected and the OVCAR-3 or A2780 cells were infected by these supernatants for 48 h. In order to obtain the cells, which were stably knocked down of ATG5 gene, the OVCAR-3 and A2780 cells were treated by 1 μM puromycin (Amresco, USA, #J593).

### Evaluation of GFP-LC3 fluorescence and tandem RFP-GFP-targeted LC3 fluorescence

In order to evaluate the level of autophagy, 5×10^4^ OVCAR-3 or A2780 cells were seeded in glass bottom cell culture dishes (code 801002, NEST, Wuxi, China). After 24 h, these cells were transfected with the plasmid of GFP-tagged LC3 plasmid (Addgene, #11546) or RFP-GFP-targeted LC3 (provided by Yoshimori) using Lipofectamine 3000 (Invitrogen, Thermo Fisher Scientific, USA) for 24 h. Subsequently, these cells were treated by vehicle or 1 μM SRT2183 for 24 h. Fluorescence images of live cells were directly taken using an inverted confocal microscope (Zeiss, Oberkochen, Germany) and 60× oil objective [35].

### Western blots

For western blots, 2 × 10^5^ OVCAR-3 or A2780 cells per well were plated in a 6-well plate for 24 h. Subsequently, the cells were incubated with the appropriate drug for distinct time periods and the western blots were performed as previously described [36] using the following antibodies: Capsese-3 (Cell Signaling Technology, USA, #9661), PARP (Cell Signaling Technology, USA, #9542), Microtubule-associated protein 1 light chain 3 (LC3) (Sigma, USA, #L7543), p62/SQSTM1/SQSTM1 (Cell Signaling Technology, USA, #5114), ATG5 (Cell Signaling Technology, USA, #9980), ATG7 (Cell Signaling Technology, USA, #8558), p-AKT (Cell Signaling Technology, USA, #9271), AKT (Cell Signaling Technology, USA, #9272), BAX (Proteintech Group, USA, #23931-1-AP), Bcl-2 (Proteintech Group, USA, #12789-1-AP), and Mcl-1 (Proteintech Group, USA, #16225-1-AP), p-p70S6K (Thr389) (Sigma, USA, #MABS82), p70S6K (Sigma, USA, #06-926), p-mTOR (S2448) (Abcam, USA, #ab109268), -S6 Ribosomal (Cell Signaling Technology, USA, #5364), S6 Ribosomal (Cell Signaling Technology, USA, #2317), mTOR (Abcam, USA, #ab2732), and β-actin (Origene, USA, #TA811000), peroxidase-linked anti-rabbit antibody (Cell Signaling Technology, Danvers, USA, #7074) or peroxidase-linked anti-mouse antibody (Sigma-Aldrich, St. Louis, USA, #A9044).

### Statistical analysis

All data were presented as mean ± standard deviation (SD) and the student’s t-test was analyzed by statistical software SPSS 22.0. *P* < 0.05 was defined as statistically significant.

## References

[1] Siegel RL, Miller KD, Jemal A. Cancer statistics, 2019. CA Cancer J Clin. 2019; 69:7–34. 10.3322/caac.21551 30620402

[2] Nick AM, Coleman RL, Ramirez PT, Sood AK. A framework for a personalized surgical approach to ovarian cancer. Nat Rev Clin Oncol. 2015; 12:239–45. 10.1038/nrclinonc.2015.26 25707631 PMC4528308

[3] Coleridge SL, Bryant A, Lyons TJ, Goodall RJ, Kehoe S, Morrison J. Chemotherapy versus surgery for initial treatment in advanced ovarian epithelial cancer. Cochrane Database Syst Rev. 2019; 2019:CD005343. 10.1002/14651858.CD005343.pub4 33543776 PMC8094177

[4] Spears N, Lopes F, Stefansdottir A, Rossi V, De Felici M, Anderson RA, Klinger FG. Ovarian damage from chemotherapy and current approaches to its protection. Hum Reprod Update. 2019; 25:673–93. 10.1093/humupd/dmz027 31600388 PMC6847836

[5] Kim JY, Cho CH, Song HS. Targeted therapy of ovarian cancer including immune check point inhibitor. Korean J Intern Med. 2017; 32:798–804. 10.3904/kjim.2017.008 28823141 PMC5583460

[6] Ventriglia J, Paciolla I, Pisano C, Cecere SC, Di Napoli M, Tambaro R, Califano D, Losito S, Scognamiglio G, Setola SV, Arenare L, Pignata S, Della Pepa C. Immunotherapy in ovarian, endometrial and cervical cancer: state of the art and future perspectives. Cancer Treat Rev. 2017; 59:109–16. 10.1016/j.ctrv.2017.07.008 28800469

[7] Lheureux S, Braunstein M, Oza AM. Epithelial ovarian cancer: evolution of management in the era of precision medicine. CA Cancer J Clin. 2019; 69:280–304. 10.3322/caac.21559 31099893

[8] Scuto A, Kirschbaum M, Buettner R, Kujawski M, Cermak JM, Atadja P, Jove R. SIRT1 activation enhances HDAC inhibition-mediated upregulation of GADD45G by repressing the binding of NF-κB/STAT3 complex to its promoter in malignant lymphoid cells. Cell Death Dis. 2013; 4:e635. 10.1038/cddis.2013.159 23681230 PMC3674366

[9] Gurt I, Artsi H, Cohen-Kfir E, Hamdani G, Ben-Shalom G, Feinstein B, El-Haj M, Dresner-Pollak R. The Sirt1 activators SRT2183 and SRT3025 inhibit RANKL-induced osteoclastogenesis in bone marrow-derived macrophages and down-regulate Sirt3 in Sirt1 null cells. PLoS One. 2015; 10:e0134391. 10.1371/journal.pone.0134391 26226624 PMC4520518

[10] Ye T, Wei L, Shi J, Jiang K, Xu H, Hu L, Kong L, Zhang Y, Meng S, Piao H. Sirtuin1 activator SRT2183 suppresses glioma cell growth involving activation of endoplasmic reticulum stress pathway. BMC Cancer. 2019; 19:706. 10.1186/s12885-019-5852-5 31319814 PMC6637499

[11] Amaravadi RK, Kimmelman AC, Debnath J. Targeting autophagy in cancer: recent advances and future directions. Cancer Discov. 2019; 9:1167–81. 10.1158/2159-8290.CD-19-0292 31434711 PMC7306856

[12] Zhang X, Schönrogge M, Eichberg J, Wendt EH, Kumstel S, Stenzel J, Lindner T, Jaster R, Krause BJ, Vollmar B, Zechner D. Blocking autophagy in cancer-associated fibroblasts supports chemotherapy of pancreatic cancer cells. Front Oncol. 2018; 8:590. 10.3389/fonc.2018.00590 30568920 PMC6290725

[13] Levy JM, Towers CG, Thorburn A. Targeting autophagy in cancer. Nat Rev Cancer. 2017; 17:528–42. 10.1038/nrc.2017.53 28751651 PMC5975367

[14] Wang J, Tan X, Yang Q, Zeng X, Zhou Y, Luo W, Lin X, Song L, Cai J, Wang T, Wu X. Inhibition of autophagy promotes apoptosis and enhances anticancer efficacy of adriamycin via augmented ROS generation in prostate cancer cells. Int J Biochem Cell Biol. 2016; 77:80–90. 10.1016/j.biocel.2016.05.020 27247025

[15] Thorburn A. Apoptosis and autophagy: regulatory connections between two supposedly different processes. Apoptosis. 2008; 13:1–9. 10.1007/s10495-007-0154-9 17990121 PMC2601595

[16] Yu L, Wan F, Dutta S, Welsh S, Liu Z, Freundt E, Baehrecke EH, Lenardo M. Autophagic programmed cell death by selective catalase degradation. Proc Natl Acad Sci USA. 2006; 103:4952–57. 10.1073/pnas.0511288103 16547133 PMC1458776

[17] Wang Y, Hu Z, Liu Z, Chen R, Peng H, Guo J, Chen X, Zhang H. MTOR inhibition attenuates DNA damage and apoptosis through autophagy-mediated suppression of CREB1. Autophagy. 2013; 9:2069–86. 10.4161/auto.26447 24189100

[18] Zhang Y, Vasheghani F, Li YH, Blati M, Simeone K, Fahmi H, Lussier B, Roughley P, Lagares D, Pelletier JP, Martel-Pelletier J, Kapoor M. Cartilage-specific deletion of mTOR upregulates autophagy and protects mice from osteoarthritis. Ann Rheum Dis. 2015; 74:1432–40. 10.1136/annrheumdis-2013-204599 24651621

[19] Kim KY, Park KI, Kim SH, Yu SN, Park SG, Kim YW, Seo YK, Ma JY, Ahn SC. Inhibition of Autophagy Promotes Salinomycin-Induced Apoptosis via Reactive Oxygen Species-Mediated PI3K/AKT/mTOR and ERK/p38 MAPK-Dependent Signaling in Human Prostate Cancer Cells. Int J Mol Sci. 2017; 18:1088. 10.3390/ijms18051088 28524116 PMC5454997

[20] Wang XY, Zhang XH, Peng L, Liu Z, Yang YX, He ZX, Dang HW, Zhou SF. Bardoxolone methyl (CDDO-me or RTA402) induces cell cycle arrest, apoptosis and autophagy via PI3K/Akt/mTOR and p38 MAPK/Erk1/2 signaling pathways in K562 cells. Am J Transl Res. 2017; 9:4652–72. 29118925 PMC5666072

[21] Eckenrode EF, Yang J, Velmurugan GV, Foskett JK, White C. Apoptosis protection by mcl-1 and bcl-2 modulation of inositol 1,4,5-trisphosphate receptor-dependent Ca2+ signaling. J Biol Chem. 2010; 285:13678–84. 10.1074/jbc.M109.096040 20189983 PMC2859530

[22] Antonsson B. Bax and other pro-apoptotic bcl-2 family “killer-proteins” and their victim the mitochondrion. Cell Tissue Res. 2001; 306:347–61. 10.1007/s00441-001-0472-0 11735035

[23] Lin L, Zhang M, Stoilov P, Chen L, Zheng S. Developmental attenuation of neuronal apoptosis by neural-specific splicing of Bak1 microexon. Neuron. 2020; 107:1180–96.e8. 10.1016/j.neuron.2020.06.036 32710818 PMC7529960

[24] Nasu Y, Benke A, Arakawa S, Yoshida GJ, Kawamura G, Manley S, Shimizu S, Ozawa T. In Situ Characterization of Bak Clusters Responsible for Cell Death Using Single Molecule Localization Microscopy. Sci Rep. 2016; 6:27505. 10.1038/srep27505 27293178 PMC4904369

[25] Leng ZG, Lin SJ, Wu ZR, Guo YH, Cai L, Shang HB, Tang H, Xue YJ, Lou MQ, Zhao W, Le WD, Zhao WG, Zhang X, Wu ZB. Activation of DRD5 (dopamine receptor D5) inhibits tumor growth by autophagic cell death. Autophagy. 2017; 13:1404–19. 10.1080/15548627.2017.1328347 28613975 PMC5584849

[26] Huang LS, Berdyshev EV, Tran JT, Xie L, Chen J, Ebenezer DL, Mathew B, Gorshkova I, Zhang W, Reddy SP, Harijith A, Wang G, Feghali-Bostwick C, et al. Sphingosine-1-phosphate lyase is an endogenous suppressor of pulmonary fibrosis: role of S1P signalling and autophagy. Thorax. 2015; 70:1138–48. 10.1136/thoraxjnl-2014-206684 26286721

[27] Yoshida GJ. Therapeutic strategies of drug repositioning targeting autophagy to induce cancer cell death: from pathophysiology to treatment. J Hematol Oncol. 2017; 10:67. 10.1186/s13045-017-0436-9 28279189 PMC5345270

[28] Aparicio S, Hidalgo M, Kung AL. Examining the utility of patient-derived xenograft mouse models. Nat Rev Cancer. 2015; 15:311–16. 10.1038/nrc3944 25907221

[29] Yoshida GJ. Applications of patient-derived tumor xenograft models and tumor organoids. J Hematol Oncol. 2020; 13:4. 10.1186/s13045-019-0829-z 31910904 PMC6947974

[30] Klionsky DJ, Abdelmohsen K, Abe A, Abedin MJ, Abeliovich H, Acevedo Arozena A, Adachi H, Adams CM, Adams PD, Adeli K, Adhihetty PJ, Adler SG, Agam G, et al. Guidelines for the use and interpretation of assays for monitoring autophagy (3^rd^ edition). Autophagy. 2016; 12:1–222. 10.1080/15548627.2015.1100356 26799652 PMC4835977

[31] Yoshii SR, Mizushima N. Monitoring and measuring autophagy. Int J Mol Sci. 2017; 18:1865. 10.3390/ijms18091865 28846632 PMC5618514

[32] Schmeisser K, Parker JA. Pleiotropic effects of mTOR and autophagy during development and aging. Front Cell Dev Biol. 2019; 7:192. 10.3389/fcell.2019.00192 31572724 PMC6749033

[33] Munson MJ, Ganley IG. MTOR, PIK3C3, and autophagy: signaling the beginning from the end. Autophagy. 2015; 11:2375–76. 10.1080/15548627.2015.1106668 26565689 PMC4835211

[34] Rafehi H, Orlowski C, Georgiadis GT, Ververis K, El-Osta A, Karagiannis TC. Clonogenic assay: adherent cells. J Vis Exp. 2011;49:2573. 10.3791/2573 21445039 PMC3197314

[35] Zhang X, Kumstel S, Jiang K, Meng S, Gong P, Vollmar B, Zechner D. LW6 enhances chemosensitivity to gemcitabine and inhibits autophagic flux in pancreatic cancer. J Adv Res. 2019; 20:9–21. 10.1016/j.jare.2019.04.006 31193017 PMC6514270

[36] Zechner D, Albert AC, Bürtin F, Vollmar B. Metformin inhibits gemcitabine induced apoptosis in pancreatic cancer cell lines. J Cancer. 2017; 8:1744–49. 10.7150/jca.17972 28819370 PMC5556636

